# A Directional Nearest Neighbor Distance-Based Algorithm for Signal Photon Extraction from Spaceborne Photon-Counting LiDAR in Shallow Waters

**DOI:** 10.3390/s26051645

**Published:** 2026-03-05

**Authors:** Shibin Zhao, Zhenwei Shi, Tingting Jin, Boxue Huang, Xiaokai Li, Hui Long

**Affiliations:** 1 Aerospace Information Research Institute, Chinese Academy of Sciences, Beijing 100094, China; zhaoshibin23@mails.ucas.ac.cn (S.Z.); shizw@aircas.ac.cn (Z.S.); jintt@aircas.ac.cn (T.J.); lixiaokai24@mails.ucas.ac.cn (X.L.); lh_lh885@139.com (H.L.); 2 Key Laboratory of Technology in Geo-Spatial Information Processing and Application System, Aerospace Information Research Institute, Chinese Academy of Sciences, Beijing 100190, China; 3 Key Laboratory of Target Cognition and Application Technology, Aerospace Information Research Institute, Chinese Academy of Sciences, Beijing 100190, China; 4 School of Electronic, Electrical and Communication Engineering, University of Chinese Academy of Sciences, Beijing 100049, China

**Keywords:** ICESat-2, photon data denoising, satellite-derived bathymetry, directional distribution

## Abstract

The Ice, Cloud, and Land Elevation Satellite-2 (ICESat-2) employs a 532 nm laser with strong water-penetration capability, making it well suited for satellite-derived bathymetry in shallow waters; however, the effective denoising of photon-counting data remains essential due to strong solar background and intrinsic instrument noise. To address this challenge, this study proposes a novel photon denoising method, termed the Directional Nearest Neighbor Distance-based Algorithm (DNNDA), for robust extraction of signal photons from shallow-water ICESat-2 data. Unlike existing methods that rely heavily on density or terrain features and often degrade under high-noise conditions, DNNDA systematically exploits both scale-corrected spatial relationships and directional distribution characteristics of photons. By quantitatively characterizing the directional features of photon distributions and embedding this information into a density representation, DNNDA amplifies the density contrast between signal and noise photons, rendering the seafloor signal photons more distinct and easier to extract. An evaluation index was further designed to automate optimal parameter determination. Validation using multiple global ICESat-2 datasets demonstrates that DNNDA achieves superior seafloor photon extraction performance, with F1-scores exceeding 95%. Further regression analysis against high-precision CUDEM data in the Puerto Rico region yields root-mean-square errors below 0.57 m. By jointly correcting scale anisotropy and incorporating directional information, DNNDA enables reliable and adaptive signal photon extraction across local and global scales, providing a robust solution for shallow-water bathymetry in complex, high-noise environments.

## 1. Introduction

As the scale and intensity of human activities in the oceans continue to expand, bathymetric data in shallow waters have become increasingly important for marine resource development, environmental protection, and maritime navigation. Conventionally, methods for acquiring bathymetric data primarily include shipborne sonar [1] and airborne Light Detection and Ranging (LiDAR) [2], both of which provide high measurement accuracy, fine spatial resolution, and relatively low noise levels. However, these approaches are time-consuming, labor-intensive, and costly, and are typically limited to relatively small spatial coverage per single cruise or flight [3]. Over the past five decades, satellite-derived bathymetry (SDB) has emerged as an effective alternative, enabling bathymetric data acquisition in areas that are difficult or impractical to access using ships or aircraft, and thereby offering a viable solution for large-scale bathymetric mapping [4,5].

To date, optical remote sensing satellites remain the primary platforms for SDB, including multispectral and hyperspectral sensors [6]. As the only spaceborne laser altimetry satellite currently in orbit with demonstrated bathymetric capability [7], ICESat-2 provides large-scale and high-precision observations for shallow-water bathymetry [8,9,10]. Its ATL03 product contains geolocated photon-level information, including longitude, latitude, and elevation, and therefore serves as a fundamental data source for SDB studies [11]. However, owing to the high sensitivity of the photon-counting instrument to solar background radiation and intrinsic system noise [12], ICESat-2 observations are often severely contaminated by noise photons. Consequently, accurate and reliable extraction of signal photons constitutes a critical prerequisite for shallow-water SDB applications.

Density-based clustering algorithms are among the most widely used for denoising ATL03 photon data [13,14,15,16,17,18,19]. They can be examined from three spatial scales: at the individual-photon scale, the nearest neighbor distance of most signal photons is generally expected to be smaller than that of noise photons. Xie et al. [20] recursively partitioned the two-dimensional space of the photon data into rectangular regions, assigning each photon a division depth representing its local spatial complexity. By applying an appropriate threshold to this value, signal photons can be effectively separated from noise photons. At the small-area scale, signal photons typically exhibit higher local density than noise photons, meaning that there are more signal photons per unit area. The Density-Based Spatial Clustering of Applications with Noise (DBSCAN) [21,22] characterizes photon density by counting neighboring photons within a predefined circular neighborhood. Photons whose local densities exceed a specified threshold, together with their neighbors, are classified as signal photons. At the overall scale, signal photons tend to exhibit spatially coherent and directional distributions, whereas noise photons are more likely to be irregularly and chaotically distributed. To better accommodate the directional behavior of signal photons, Zhang et al. [23] modified DBSCAN by replacing the circular neighborhood with an elliptical one. Additionally, Leng et al. [24] introduced an extended elliptical search region beyond the original neighborhood to identify the most probable distribution direction of signal photons. Although these algorithms have demonstrated effectiveness to varying degrees, several limitations remain. Algorithms operating at the individual-photon scale may misclassify noise photons with small nearest neighbor distances as signal photons, leading to reduced recognition accuracy, particularly in high-noise environments. Approaches that consider small-area density often yield indistinct signal boundaries, while algorithms working at the overall scale typically rely on preset directional parameters, limiting their adaptability to complex terrain and high-noise scenarios. These observations indicate that existing algorithms do not fully exploit the directional characteristics inherent in photon distributions, motivating this study to enhance the utilization of directional features in order to improve signal photon recognition accuracy, particularly under high-noise-rate conditions.

In this study, a specialized signal photon extraction algorithm specifically designed for shallow-water environments is developed. The proposed method first segments raw ATL03 photon data, then quantifies the density characteristics of photons, and employs an iterative strategy for photon extraction. This method effectively clusters spatially correlated photons by leveraging the intrinsic distribution properties of the photon data, rather than relying on preset density or terrain assumptions. Comprehensive validation across six representative global study areas demonstrates that the proposed approach can robustly and reliably extract both shallow-ocean seafloor and sea-surface signal photons under diverse topographic and noise conditions.

## 2. Materials and Methods

### 2.1. Materials

#### 2.1.1. ICESat-2/ATLAS

ICESat-2 is an Earth observation satellite launched by National Aeronautics and Space Administration (NASA) in September 2018 and represents the first Earth-orbiting mission equipped with a photon-counting LiDAR system. Its primary objective is to quantify elevation changes of the Earth’s surface—particularly over polar ice sheets and glaciers—which is essential for monitoring and understanding global climate change [25,26].

The core instrument onboard ICESat-2 is Advanced Topographic Laser Altimeter System (ATLAS), a laser ranging system that estimates surface elevation by measuring the round-trip travel time of returned photons. ICESat-2/ATLAS operates at an altitude of approximately 500 km and emits laser pulses at a wavelength of 532 nm with a repetition frequency of 10 kHz [12,27]. Along the ground track, these pulses generate overlapping footprints with an approximate diameter of 10 m at a spacing of about 0.7 m. Each transmitted pulse is split into three beam pairs aligned along the flight direction, where each pair consists of one strong beam and one weak beam [28].

In this study, we use the ICESat-2 ATL03 and ATL24 global geolocated photon data products. The ATL03 provides along-track photon locations and associated attributes at single-photon precision, and is a latest level 3A product based on ATL03 [29], employing integrated machine learning algorithms for photon signal labelling. Both datasets can be accessed and downloaded from Earthdata Search portal of NASA “https://search.earthdata.nasa.gov/search” (accessed on 4 October 2025).

#### 2.1.2. Reference DEM Data

The Continuously Updated Digital Elevation Model (CUDEM) is an integrated coastal elevation infrastructure initiative led by the United States National Centers for Environmental Information (NCEI) [30]. It is designed to provide seamless bare-earth, topography–water depth, and bathymetric Digital Elevation Model (DEM) products covering the U.S. Atlantic and Gulf coasts, Hawaii, U.S. territories, and portions of the Pacific coast. CUDEM data can be accessed and downloaded from the National Oceanic and Atmospheric Administration (NOAA) Data Access Viewer “https://coast.noaa.gov/dataviewer/#/lidar/search” (accessed on 26 October 2025).

The data sources used to generate CUDEM are heterogeneous, and when multiple datasets overlap, CUDEM assesses the relative quality of each source using visual inspection and independent measurements, typically removing older and less accurate datasets to minimize errors in the final DEM. CUDEM is currently one of the most detailed coastal bathymetric datasets in North America, with a spatial resolution of 1/9 arc-second (≈3 m), while the nearshore bathymetric DEMs have a resolution of 1/3 arc-second (≈10 m). According to the CUDEM documentation [30], the dataset provides a nominal vertical accuracy of ~50 cm and horizontal accuracy of ~100 cm.

CUDEM is georeferenced to the North American Datum of 1983 (NAD83), and elevations are reported in meters and referenced to Puerto Rico Vertical Datum 2002 (PRVD02). In contrast, ICESat-2 geolocation is based on WGS84 (incorporating ITRF2014) and elevations are provided as WGS84 ellipsoidal heights, which differ from the CUDEM horizontal and vertical datums. Therefore, the NOAA elevation conversion tool is employed to transform CUDEM elevations and ensure datum consistency between the two datasets.

#### 2.1.3. Study Areas

**Figure 1:**
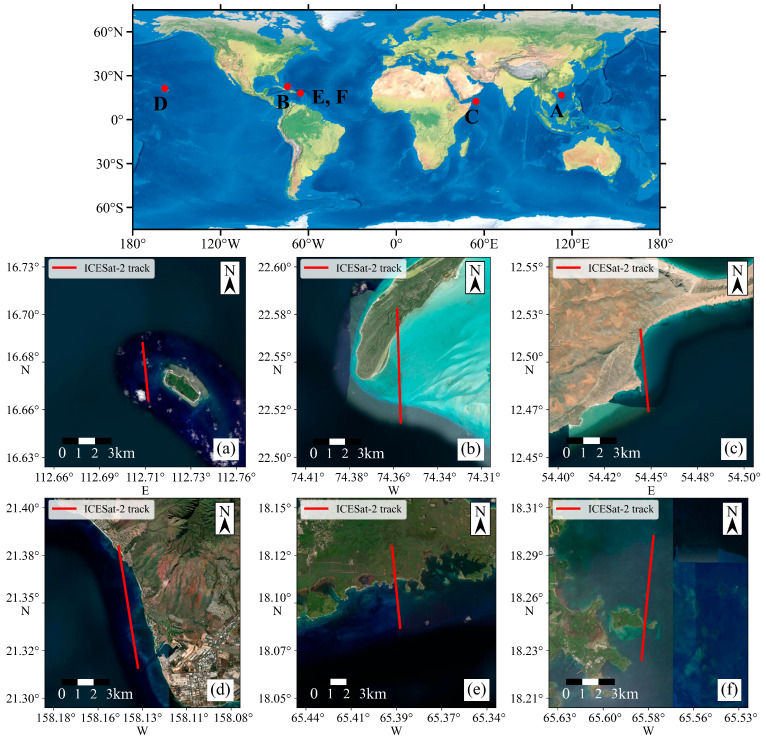
Distribution of the study areas. Panels (**a**–**f**) show the specific distribution of the datasets used in this study.

Six ICESat-2 datasets from five different sea areas were used in this study. As shown in Figure 1, dataset A is located near the East Island of the Xisha Islands; dataset B is located near Long Island in the shallow lagoon of Acklins Bay, Bahamas; dataset C is located off the east coast of Socotra Island at the junction of the Arabian Sea and the Gulf of Aden; dataset D is located near the Hawaiian Islands. The above four areas have diverse topographic and geomorphologic settings, which can effectively reflect the performance of the proposed algorithm. Meanwhile, to further validate the accuracy of the extracted photons, two datasets (E, F) near Puerto Rico are used to compare the signal extraction results with high-precision DEMs in the study area. Table 1 lists the specific information of each dataset.

### 2.2. Methods

The relative density and directional distribution of signal photons are key characteristics that can vary substantially across different areas. This variability reduces the effectiveness and general applicability of methods that rely on preset density thresholds and photon distribution directions. To address this issue, we propose a signal photon extraction method that leverages both the spatial density and directional distribution characteristics inherent in photon data.

**Figure 2:**
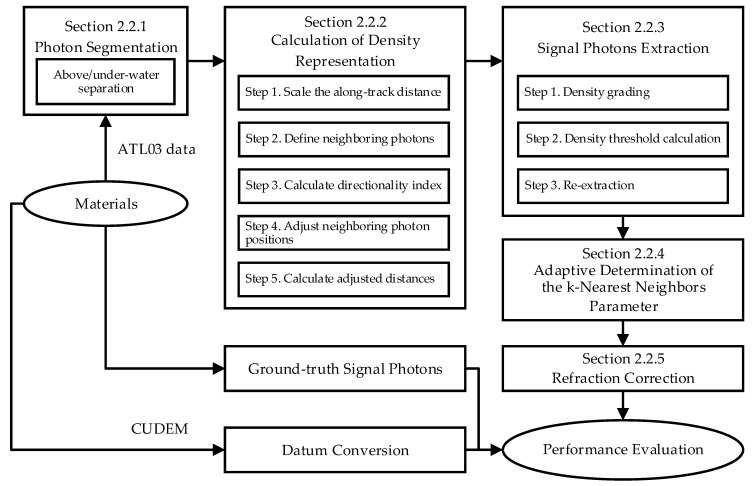
Workflow of this study.

Figure 2 illustrates the main workflow of this study, which consists of three key components: (1) Data preprocessing phase, where raw ATL03 data undergoes photon segmentation based on the along-elevation density distribution, thereby separating the above-water and underwater photon subspaces. (2) Directional density feature computation—after distance scaling, the directional indices of neighboring photons and their distances from the central photon are calculated; through direction-aware distance adjustment and aggregation, a density representation incorporating directional characteristics is obtained. (3) Density threshold determination for signal photon extraction, coupled with iterative secondary extraction to identify surface, land, and seabed photons. Additionally, to enhance the adaptability of proposed algorithm, this study designed metrics for identifying optimal parameters. Finally, the extraction performance of the proposed algorithm is systematically evaluated and validated.

#### 2.2.1. Photon Segmentation

**Figure 3:**
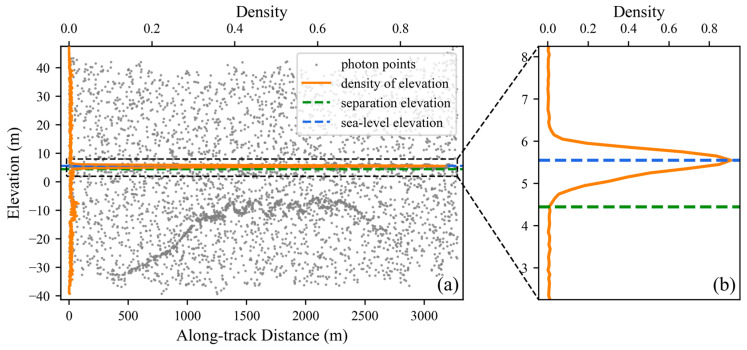
Along-elevation density distribution of the photon point cloud. Panel (**a**) shows the full view. Panel (**b**) shows the zoomed-in view.

Considering the attenuation effect of water on the laser signal, the spatial density of underwater signal photons is significantly lower than that of above-water signal photons. To prevent underwater signal photons from being misidentified as noise during the denoising process, we determine a separation elevation to distinguish water-surface photons from underwater photons. Specifically, we employ elevation histogram statistics to characterize the along-elevation density distribution and to identify sea-level photons, and then partition the photon data into above-water and underwater segments using the separation elevation, defined as the first local minimum of the longitudinal density distribution below the sea-level elevation. Furthermore, since this study focuses on shallow-water scenarios, we consider only photons within 100 m above and below the sea-level elevation. Experiments show that the distinct density peak formed by sea-level photons along the elevation direction provides a reliable basis for separating above-water and underwater photons (Figure 3).

#### 2.2.2. Calculation of Density Representation

Underwater signal photons are not only characterized by higher local density than noise photons, but also by exhibiting more structured and directional spatial patterns. As shown in Figure 2, to capture these characteristics, we analyze the spatial configuration of neighboring photons and quantify the degree of anisotropy in their local distribution. By measuring how strongly the neighboring photons are aligned along a predominant direction, we obtain a directional descriptor that reflects the underlying structure of the photon field.

This directional metric is then incorporated into the computation of the k-nearest neighbor distances, effectively refining the local density representation. In this way, photon clusters with clear geometric structure can be distinguished from more isotropic and randomly distributed noise photons, which enhances the robustness of signal photon identification. The detailed procedure is as follows.

Step 1. Scale the along-track distance:

To effectively utilize density features at the overall scale, it is necessary to ensure that the neighborhood of each photon contains sufficient global structural information. Therefore, the along-track distances of the photon point cloud are compressed so that the neighborhood constructed for each photon covers a relatively larger extent of the overall structure. Based on empirical observations, the along-track distance of each photon is multiplied by a scaling coefficient of 0.025. This correcting scale allows each photon neighborhood to incorporate more overall-scale density characteristics in the subsequent analysis.

**Figure 4:**
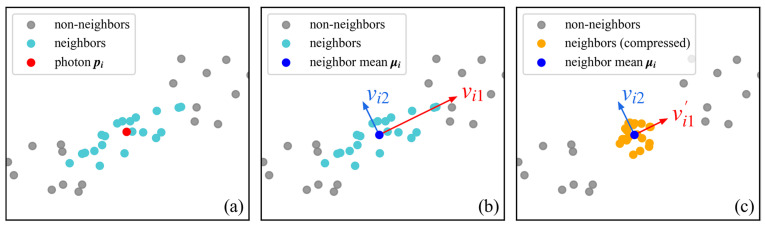
Illustration of neighborhood photon compression. Panel (**a**) shows the neighboring photons. Panel (**b**) shows the eigenvectors. Panel (**c**) shows the compressed neighbors.

Step 2. Define neighboring photons:

Let each photon in the photon space be represented by a two-dimensional coordinate vector as:

(1) $$p_{i}={\left[x_{i},h_{i}\right]}^{T},i=1,2,\cdots ,n$$

where $x_{i}$ represents the scaled along-track distance, $h_{i}$ represents elevation. This yields the vectors $\left\{p_{1},p_{2},\ldots ,p_{i},\ldots ,p_{n}\right\}$. For each photon $p_{i}$ in the dataset, find its k nearest neighbors (Figure 4a), denoted as $\left\{p_{i1},p_{i2},\ldots ,p_{ij},\ldots ,p_{ik}\right\}$, where $p_{ij}$ denotes the j-th neighboring photon of $p_{i}$.

Step 3. Calculate directionality index:

For each photon $p_{i}$, first compute its neighborhood photon average $\mu_{i}$, i.e., calculate the mean values of all neighborhood photons along-track coordinate and along-elevation coordinate, respectively. Then construct the local covariance matrix using its k nearest neighbors:

(2) $$C_{i}=\frac{1}{k}\sum_{j=1}^{k}\left(p_{ij}-\mu_{i}\right){\left(p_{ij}-\mu_{i}\right)}^{T}$$

Since $p_{ij}\in R^{2\times 1}$, $C_{i}$ is a $R^{2\times 2}$ real symmetric matrix.

For each photon $p_{i}$, performing an eigenvalue decomposition on its covariance matrix $C_{i}$. yields the corresponding eigenvalues $\lambda_{i1}\geq \lambda_{i2}>0$ and eigenvectors $v_{i1}$, $v_{i2}$, where $v_{i1}$ represents the first principal component direction of the local neighborhood of $p_{i}$ (Figure 4b). To quantify the directionality of the spatial distribution of neighboring photons and to control the subsequent adjustment, we define a directionality-based scaling factor $\beta_{i}$ as:

(3) $$\beta_{i}=\sqrt{\frac{\lambda_{i2}}{\lambda_{i1}}},\;0<\beta_{i}\leq \;1$$

when $\lambda_{i1}>\lambda_{i2}$, the neighborhood is more elongated along the first principal component $v_{i1}$, and $\beta_{i}<1$. A smaller $\beta_{i}$ indicates stronger directionality and implies stronger compression along $v_{i1}$ in the subsequent adjustment step.

Step 4. Adjust neighboring photon positions:

Based on the directionality-based scaling factor $\beta_{i}$, we adjust the positions of neighboring photons to account for the influence of locally directional structure on their spatial distance from photon $p_{i}$.

In the principal component coordinate system, only the first component is scaled by $\beta_{i}$, while the second component remains unchanged. This projection–scaling back–projection procedure can be equivalently expressed as a single linear transformation in the original coordinate system. For any neighboring photon $p_{ij}$, its adjusted position $p_{ij}^{\prime }$ is given by

(4) $$p_{ij}^{\prime }=\mu_{i}+V_{i}B_{i}V_{i}^{T}\left(p_{ij}-\mu_{i}\right)$$

where $B_{i}=diag\left(\beta_{i},\;1\right)$ is the diagonal scaling matrix that scales only the first principal component, and $V_{i}=\left[v_{1},\;v_{2}\right]$ is the eigenvector matrix of $C_{i}$.

Geometrically, this transformation is equivalent to first projecting $\left(p_{ij}-\mu_{i}\right)$ onto the basis spanned by $v_{i1}$ and $v_{i2}$, then scaling the component along the first principal component direction by a factor of $\beta_{i}$ while keeping component along the second component unchanged, and finally mapping the result back into the original coordinate system. In this way, the dispersion along the first principal component direction is compressed, enabling directionality to be reflected in subsequent distance calculations (Figure 4c).

Step 5. Calculate adjusted distances:

After obtaining the adjusted positions $p_{ij}^{\prime }$, we compute the new Euclidean distance between each neighboring photon and the photon $p_{i}$:

(5) $$d_{ij}^{\prime }=\left|\left|p_{ij}^{\prime }-p_{i}\right|\right|$$

where   denotes the Euclidean distance. Compared with the original distance $d_{ij}=\left|\left|p_{ij}-p_{i}\right|\right|$ the adjusted distance $d_{ij}^{\prime }$ explicitly incorporates the influence of directional structure on the spatial relationship between photons.

Finally, for each photon $p_{i}$, we sum the adjusted distances of its k nearest neighbors:

(6) $$D_{i}=\sum_{j=1}^{k}d_{ij}^{\prime }$$

where $D_{i}$ is a density representation of the central photon $p_{i}$ that accounts for directional effects. The smaller the value of $D_{i}$, the greater the density of $p_{i}$; conversely, the larger the value of $D_{i}$, the smaller the density of $p_{i}$.

#### 2.2.3. Signal Photons Extraction

The photon density representation is a key indicator for distinguishing signal photons from noise photons. As shown in Figure 2, based on the density measure $D_{i}$ defined above, we determine a density boundary that separates signal photons from noise photons. This is achieved by grading the density values and automatically computing an optimal density threshold. In addition, to further improve the reliability of underwater signal photon extraction, we apply a secondary refinement (re-extraction) to the coarsely classified signal photons. The detailed procedure is as follows.

Step 1. Density grading:

First, we compute the range of density values $\left\{D_{i}\right\}$ over the entire dataset and obtain the minimum and maximum values, $D_{min}$ and $D_{max}$. This range is then divided into M density grades using linear binning. The bin width is defined as

(7) $$width_{bin}=\frac{D_{max}-D_{min}}{M}$$

where M is the total number of grades. Each photon $p_{i}$ is assigned to a density grade $G_{i}$ according to:

(8) $$G_{i}=floor\left(\frac{D_{i}-D_{min}}{width_{bin}}\right)+1$$

where $floor\left(number\right)$ denotes the greatest integer less than or equal to number, $width_{bin}$ is the uniform width of each density grade.

Step 2. Density threshold calculation:

To separate signal photons from noise photons based on their density grades, we determine an optimal grade threshold using the Otsu method [31,32]. This method selects the threshold that maximizes the between-class variance between two groups.

We construct a histogram of the discrete grades $\left\{G_{i}\right\}$ and normalize it to obtain the probability of each grade. For a candidate threshold T, the photons are divided into two classes:

(9) $$\left\{\begin{array}{c} signal:\;G_{i}\leq T\\ noise:\;G_{i}>T \end{array}\right.$$

Let the $\omega_{1}\left(T\right)$ and $\omega_{2}\left(T\right)$ denote the weights of the signal and noise classes; $\mu_{1}\left(T\right)$ and $\mu_{2}\left(T\right)$ denote their mean grades, respectively, the between-class variance $\sigma^{2}\left(T\right)$ for threshold T is given by

(10) $$\sigma^{2}\left(T\right)=\omega_{1}\left(T\right)\omega_{2}\left(T\right){\left[\mu_{1}\left(T\right)-\mu_{2}\left(T\right)\right]}^{2}$$

We evaluate $\sigma^{2}\left(T\right)$ for all possible thresholds T=1,2,⋯,M, and select the threshold that maximizes the between-class variance $T_{opt}$:

(11) $$T_{opt}=\underset{1\leq T\leq M}{argmax}\sigma^{2}\left(T\right)$$

Photons with $G_{i}\leq T_{opt}$ are retained as candidate signal photons, while those with $G_{i}>T_{opt}$ are classified as noise.

Step 3. Re-extraction:

To further improve the accuracy of signal photon extraction, we perform a secondary extraction on the candidate signal photons obtained in Step 2. In this stage, the density characteristics are re-evaluated within the subset of candidate signal photons using exactly the same parameters and procedure as in the initial extraction. A new density threshold is computed on this subset, and photons that do not satisfy this refined threshold are discarded (from Step 2 in Section 2.2.2 to Step 2 in Section 2.2.3). This iterative refinement helps reduce the number of noise photons that were misclassified as signal photons in the initial extraction.

#### 2.2.4. Adaptive Determination of the k-Nearest Neighbors Parameter

In the proposed algorithm, the number of nearest neighbors, k, is a sensitivity parameter that requires adjustment based on the dataset. To enhance the adaptivity of the algorithm, this study designs an index, based on prior distribution of ideal signal photons, to evaluate the continuity and width of extracted signal photons. By comparing the values of this index, the optimal number of nearest neighbors k can be efficiently determined.

**Figure 5:**
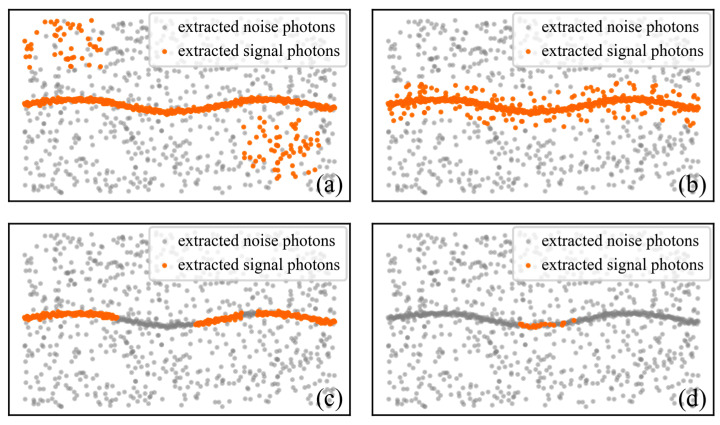
Typical patterns of unsatisfactory extraction performance. Panel (**a**) shows a large number of photons being misidentified. Panel (**b**) shows the broader width of the signal photon. Panel (**c**) shows the signal photon exhibiting interruption. Panel (**d**) shows the extraction being overly conservative.

For the extraction of signal photons from any given ATL03 dataset over a specific shallow-water area, the ideally extracted signal photons should exhibit the following properties: the signal photons should be continuous and smooth, and noise photons should not be mistakenly identified as signal photons; the width of the signal photon should be as slim as possible and clearly separated from noise photons; at the same time, all the true signal photons present in the data should be identified as completely as possible. In contrast, four typical patterns correspond to unsatisfactory extraction performance: (1) a large number of noise photons are misclassified as signal photons (Figure 5a); (2) noise photons adjacent to the signal photons are incorrectly classified as signal photons, which leads to a broader extraction result (Figure 5b); (3) the signal photons are not continuous and exhibit obvious gaps (Figure 5c); (4) the extraction is overly conservative, resulting in an insufficient number of valid signal photons (Figure 5d).

Based on these observations, the two parts of the index are designed as follows. The first part is a continuity index, which focuses on the continuity of signal photons and the accuracy of their extraction. The specific procedure is as follows:

Step 1. Quantifying the elevation variation of extracted signal photons:

The normal along-track gap of ATL03 photon data is 0.7 m. The extracted signal photons are therefore clustered according to identical along-track coordinates, and the mean elevation within each cluster is computed, yielding a new point sequence $\left\{\left(x_{i},h_{mean\left(i\right)}\right)\right\},i=1,2,\cdots ,N_{count}$, where $x_{i}$ denotes the along-track coordinate of the i-th cluster, $h_{mean\left(i\right)}$ denotes the mean elevation of photons within that cluster, and $N_{count}$ is the total number of signal-photon clusters. The sum of squared first-order differences in elevation for this sequence is then computed as:

(12) $$S_{1}=\sum_{i=1}^{N_{count}-1}{\left(h_{mean\left(i+1\right)}-h_{mean\left(i\right)}\right)}^{2}$$

where the quantity $S_{1}$ characterizes, to some extent, the smoothness of the extracted signal photons. If noise photons are misclassified as signal photons, the mean elevation of the affected cluster may deviate abnormally from that of neighboring signal-photon clusters, increasing ${\left(h_{mean\left(i+1\right)}-h_{mean\left(i\right)}\right)}^{2}$ and consequently leading to a larger value of $S_{1}$.

Step 2. Penalizing interruptions in extracted signal photons:

In this study, all along-track gaps between signal-photon clusters are examined. If the gap exceeds 70 m (Equivalent to the loss of 100 clusters of signal photons), it is regarded as an abnormal interruption and is penalized. The total penalty $S_{2}$ is computed as:

(13) $$S_{2}=\sum_{j=1}^{N_{penalty}}\left(N_{step\left(j\right)}\times h_{range}^{2}\right)$$

where $N_{penalty}$ is the number of interruptions that receive a penalty, $h_{range}$ is the vertical range of the photon data, and $N_{step\left(j\right)}$ is the number of missing photon clusters within the j-th interruption. The latter is calculated as $N_{step}=\frac{\Delta x}{0.7}-1$, where Δx denotes the along-track distance between the two clusters at the ends of the interruption.

Step 3. Computing the continuity index:

After quantifying both the smoothness and continuity of the extracted signal photons, the continuity index $index_{1}$ is defined as:

(14) $$index_{1}=\frac{S_{1}+S_{2}}{r_{signal}}$$

where $r_{signal}$ is the ratio of the number of extracted signal photons to the total number of photons. Dividing by $r_{signal}$ prevents extremely conservative extraction results (e.g., only a single signal photon being extracted) from receiving an artificially favorable score. A smaller value of $index_{1}$ indicates, to some extent, better smoothness and continuity of the extracted signal photons.

The second part is a sharpness index, which reflects the width of the signal photons. It is computed as:

(15) $$index_{2}=\frac{\sum_{i=1}^{N_{count}}\sigma_{i}^{2}}{N_{count}\times r_{signal}}$$

where $N_{count}$ is the total number of signal-photon clusters, $\sigma_{i}^{2}$ denotes the variance of the elevation coordinates of photons within the i-th cluster, and $r_{signal}$ is again the ratio of the number of extracted signal photons to the total number of photons, used to avoid overly conservative extraction results. A smaller value of $index_{2}$ indicates, to some extent, slimmer width of the extracted signal photons.

Finally, the overall index used in this study for parameter evaluation is obtained by multiplying the two parts:

(16) $$index=index_{1}\times index_{2}$$

Within the same dataset, a smaller value of index indicates, to some extent, better performance of signal-photon extraction under the corresponding parameter setting. Therefore, for a given dataset, the optimal parameter k can be efficiently selected by comparing the index values associated with different choices of k.

#### 2.2.5. Refraction Correction

The photon elevations in ATL03 have already been corrected for factors such as atmospheric delay and systematic errors. However, bathymetric errors caused by light refraction at the air–water interface and within the water column are typically not included in these corrections. The magnitude of refraction effects is strongly influenced by water properties, and variations in water quality can therefore lead to site-dependent refraction-induced biases in photon-based bathymetry. To this end, based on research by Parrish and Shi et al. [16,33], we correct the elevation of underwater photons using the following correction equation:

(17) $$h=h_{0}+0.25416\times \Delta h$$

where h represents the corrected bathymetric photon elevation, $h_{0}$ represents the original elevation, and Δh represents the water depth from sea surface to the bathymetric photon.

#### 2.2.6. Evaluation Methods for Algorithm Performance

Following the aforementioned procedure to obtain signal photons, it is necessary to further validate the effectiveness of the algorithm. In this study, manually labeled signal photons are regarded as ground-truth signal photons. Ground-truth selection was performed by researchers by jointly referencing the confidence labels from ATL03, the classification labels from ATL08, and visual interpretation. Manual labeling was conducted using PhotonLabeling, a custom-developed interactive software designed for ICESat-2 photon data annotation (publicly available for download at: https://github.com/zwshi-pku/PhotonLabeling). These ground-truth signal photons are used to compute the precision (P), recall (R), and F1-score of the extracted results. Precision represents the proportion of true signal photons among the extracted signal photons, whereas recall denotes the proportion of ground-truth signal photons that are correctly identified by the algorithm. The F1-score is the harmonic mean of precision and recall. The formulas for calculating these metrics are as follows:

(18) $$P=\frac{TP}{TP+FP}$$

(19) $$R=\frac{TP}{TP+FN}$$

(20) $$F=\frac{2P\cdot R}{P+R}$$

where TP represents the number of correctly detected signal photons, FP is the number of erroneously detected signal photons, and FN is the number of noise photons incorrectly detected as signal photons.

The extraction results for areas E and F are validated using the CUDEM from the Puerto Rico area. Subsequently, regression analysis is performed on the extracted signal photon elevations and the corresponding CUDEM elevations, with evaluation metrics including the coefficient of determination ($R^{2}$), RMSE (root-mean-square error), and MAE (mean absolute error) calculated to assess the model’s performance.

To illustrate the effectiveness of our approach, we compare it with two established algorithms, namely DBSCAN [21,22] and HDWC (Heterogeneous Density and Weak Connectivity) [17]. For fair comparison, the parameters of both reference methods were tuned according to settings reported in the corresponding literature, ensuring that each method operates under its recommended configuration. Additionally, photon classification labels from the ATL24 bathymetry product have been incorporated into the comparison framework. Because the primary focus of this study is the extraction of underwater signal photons, the evaluation exclusively uses underwater photon data, allowing a direct comparison of performance on the target task.

## 3. Results

### 3.1. Parameters Settings

The parameters involved in this study can be categorized into two types: non-sensitive parameters, which use default values across all datasets; and sensitive parameters, which require adjustment based on the dataset. Non-sensitive parameters include the bin size used for histogram statistics of along-elevation density (Section 2.2.1), the scaling coefficient to scale along-track distance (Section 2.2.2) and the total number of grades M used for density classification (Section 2.2.3). Based on empirical tests, the bin size in this study is set to 0.1, which approximates the vertical accuracy of ICESA-2/ATLAS [12]. The scaling coefficient is set to 0.025, and the number of levels M is set to 20.

**Figure 6:**
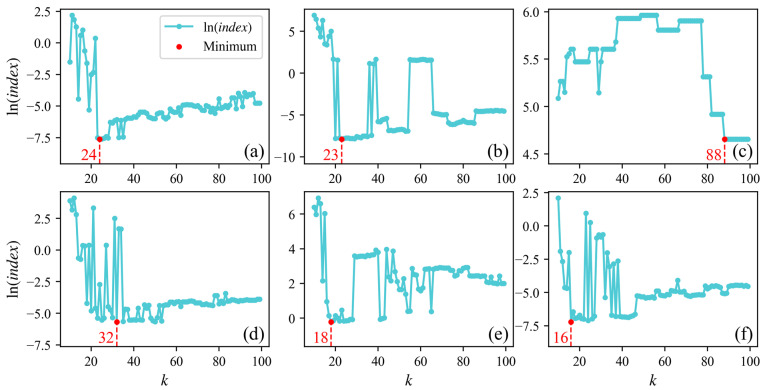
Logarithms of indices calculated using different k in above-water photons. Panels (**a**–**f**) show results for areas A–F.

**Figure 7:**
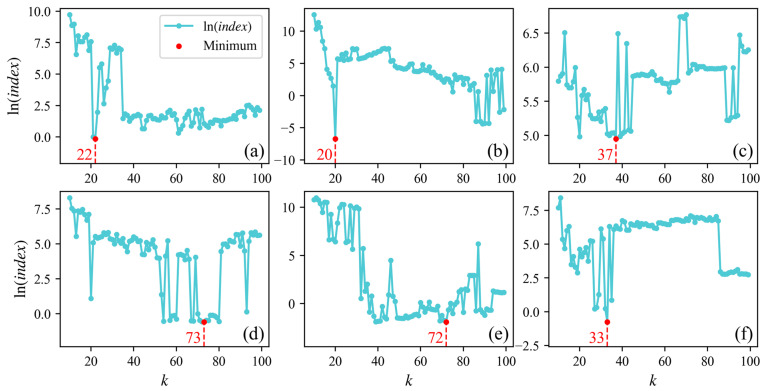
Logarithms of indices calculated using different k in in underwater photons. Panels (**a**–**f**) show results for areas A–F.

The proposed method involves a sensitive parameter: the number of neighboring points k. The range of candidate values for k selected in this study is 10–100 (where a too-small k hinders the calculation of the directionality of neighboring photons, while a too-large k reduces the distinguishability between photons). By calculating the minimum value of the signal photon extraction effectiveness indices in Section 2.2.4, we can rapidly determine the optimal k for the six experimental areas in this study. Figure 6 and Figure 7 respectively illustrate the relationship between the logarithm values of indices and parameter k for the above-water and underwater sections. Table 2 presents the values of k for each area.

### 3.2. Extraction Results of Signal Photons

**Figure 8:**
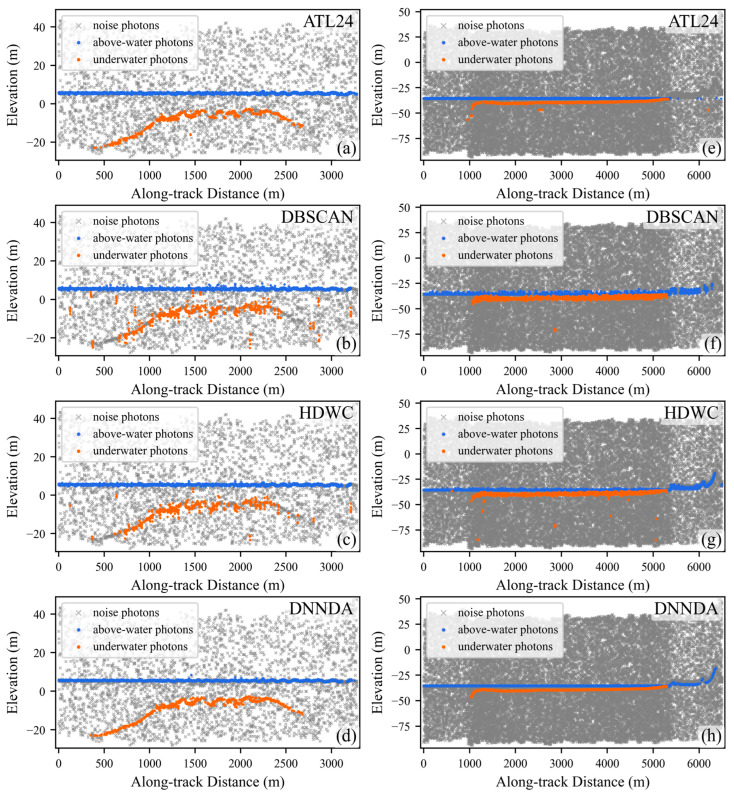
Extraction results of the three algorithms in areas A and B. Panels (**a**–**d**) show area A. Panels (**e**–**h**) show area B.

Figure 8 illustrates the extraction performance of the four algorithms in areas A and B. In area A, DBSCAN misclassifies some isolated noise photons as signal photons, while HDWC exhibits weaker signal continuity compared to the proposed DNNDA algorithm; ATL24 demonstrates signal photon characteristics relatively similar to DNNDA. In area B, all four algorithms demonstrate satisfactory extraction performance. However, ATL24 identifies fewer isolated signal photons, while DBSCAN and HDWC detect signal photons with broader widths. The proposed DNNDA algorithm clearly distinguishes signal photons from noise photons.

**Figure 9:**
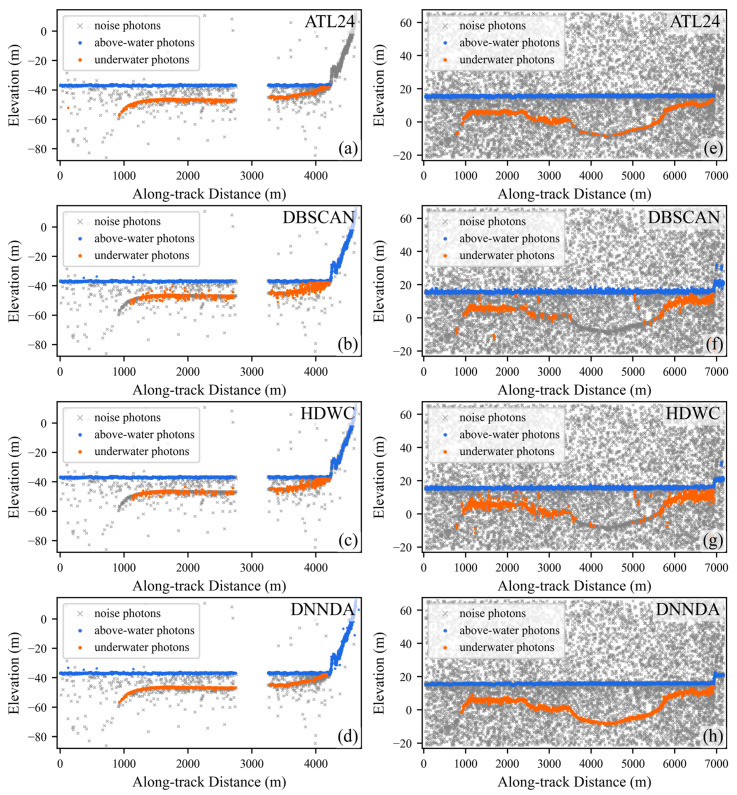
Extraction results of the three algorithms in areas C and D. Panels (**a**–**d**) show area C. Panels (**e**–**h**) show area D.

Figure 9 displays the extraction results for areas C and D. Area C exhibits less noise, and all four methods successfully extracted the primary signal photons. However, due to the lack of distance scaling and the absence of direction-aware feature extraction for signal photons, both DBSCAN and HDWC suffer from poor continuity in their extracted signal photons. In contrast, the proposed DNNDA method extracts signal photons with good continuity and narrower width. Area D data contains substantial background noise and exhibits greater seafloor depth. DBSCAN and HDWC produces several false isolated detections, with some seafloor regions undetected. ATL24 exhibits poor continuity of signal photons in deeper water. In contrast, DNNDA-extracted signal photons demonstrate superior continuity alongside narrower width.

**Figure 10:**
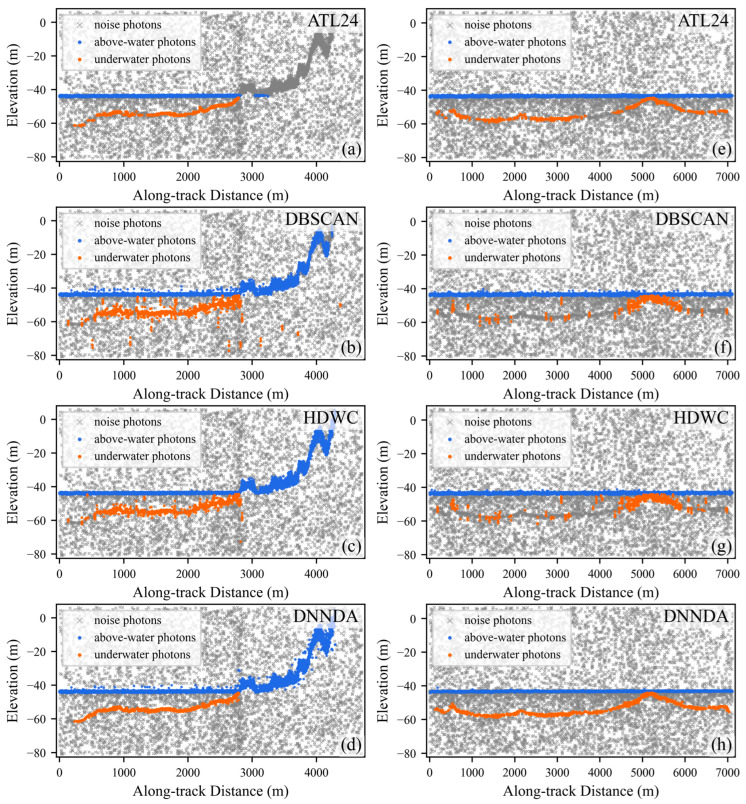
Extraction results of the three algorithms in areas E and F. Panels (**a**–**d**) show area E. Panels (**e**–**h**) show area F.

Figure 10 displays the extraction performance in areas E and F. In area E, the proposed DNNDA significantly demonstrates slimmer signal photon width and better continuity of signal photons, particularly within the 0–1000 m along-track distance range, where DBSCAN and HDWC exhibited poor underwater signal photon extraction performance. In area F, where the distinction between underwater signal photons and noise photons is low. The proposed DNNDA still achieves relatively robust extraction performance in this challenging case. In contrast, the other two algorithms extract only a limited number of signal photons near the water surface. Due to the lack of compensation for scale differences between the along-track and along-elevation dimensions, the signal photons extracted by these two algorithms exhibit a pronounced along-elevation pattern, which is inconsistent with the typical orientation of land-related features. Meanwhile, ATL24 shows poor continuity in signal photon extraction across both areas, with multiple interruptions occurring.

### 3.3. Evaluation of Signal Photon Extraction Accuracy

The performance of underwater signal photon extraction by the ATL24, DBSCAN, HDWC, and the proposed DNNDA was evaluated through a comparison with manually selected photons, with accuracy metrics including precision, recall, and F1-score calculated and presented in Table 3. The results demonstrate a clear superiority of the proposed DNNDA across all experimental areas. For instance, in area A, the proposed algorithm achieves a precision of 97.38%, a recall of 94.50%, and an F1-score of 95.92%, significantly outperforming the DBSCAN (P 70.29%, R 62.06%, F 65.92%) and the HDWC (P 76.41%, R 63.05%, F 69.09%). This trend of superior performance is consistent across other areas, such as area B, where the proposed DNNDA records a precision of 99.15%, a recall of 97.37%, and an F1-score of 98.25%, compared to much lower values for DBSCAN (P 78.27%, R 77.47%, F 77.87%) and HDWC (P 81.56%, R 79.66%, F 80.60%).

Notably, in challenging areas like D and F, where width of extracted signal photons is often broader and signals appear discontinuous with numerous isolated points, the proposed algorithm maintains high accuracy. In area D, the proposed DNNDA achieves a precision of 96.49%, a recall of 99.08%, and an F1-score of 97.77%, while DBSCAN and HDWC show notably lower metrics (DBSCAN: P 88.45%, R 53.51%, F 66.68%; HDWC: P 84.65%, R 71.44%, F 77.49%). Similarly, in area F, the proposed DNNDA records a precision of 96.62%, a recall of 98.14%, and an F1-score of 97.37%, far exceeding the performance of DBSCAN (P 76.21%, R 41.70%, F 53.91%) and HDWC (P 72.18%, R 45.85%, F 56.08%).

ATL24 also performs well across the experimental areas, except for area F where the F1-score is only 78.52%—due to long interruptions in the extracted signal photons. In all other areas, the F1-score exceeds 85%.

These results are visually consistent with the extraction performance discussed in Section 3.2, where areas with poor accuracy and signal continuity correspond to lower precision and recall for DBSCAN and HDWC. In contrast, the proposed DNNDA consistently delivers high-quality signal extraction with slimmer width and improved continuity, even under complex topographic conditions.

### 3.4. Evaluation of Bathymetric Accuracy

By comparing the performance metrics of ATL24, DBSCAN, HDWC, and the proposed DNNDA across areas E and F, the bathymetric accuracy of each algorithm can be effectively evaluated. These metrics were obtained through regression analysis after applying a vertical datum transformation to the extracted signal photon elevations and comparing them with the corresponding elevations from the CUDEM dataset, thereby characterizing the bathymetric performance of each algorithm.

Table 4 reveals that the proposed DNNDA exhibits significant advantages over both DBSCAN and HDWC in the two test areas. For example, in area E, the proposed method achieves an R^2^ value of 0.98, with RMSE and MAE values of 0.45 m and 0.31 m, respectively, thereby clearly outperforming DBSCAN (R^2^ = 0.14, RMSE = 5.44 m, MAE = 1.45 m) and HDWC (R^2^ = 0.73, RMSE = 1.82 m, MAE = 0.68 m). The poor performance of DBSCAN in this area, reflected by its low R^2^ and large error metrics, indicates substantial misidentification of noise photons as signal photons and consequently very low bathymetric precision.

In area F, the proposed DNNDA remains evident, attaining an R^2^ value of 0.99, with RMSE and MAE of 0.56 m and 0.40 m, respectively; these results are substantially better than those for DBSCAN (R^2^ = 0.87, RMSE = 1.40 m, MAE = 0.78 m) and HDWC (R^2^ = 0.76, RMSE = 1.93 m, MAE = 1.04 m). This consistent performance across both areas suggests that the proposed DNNDA maintains high precision and stability under different geographic and noise conditions.

**Figure 11:**
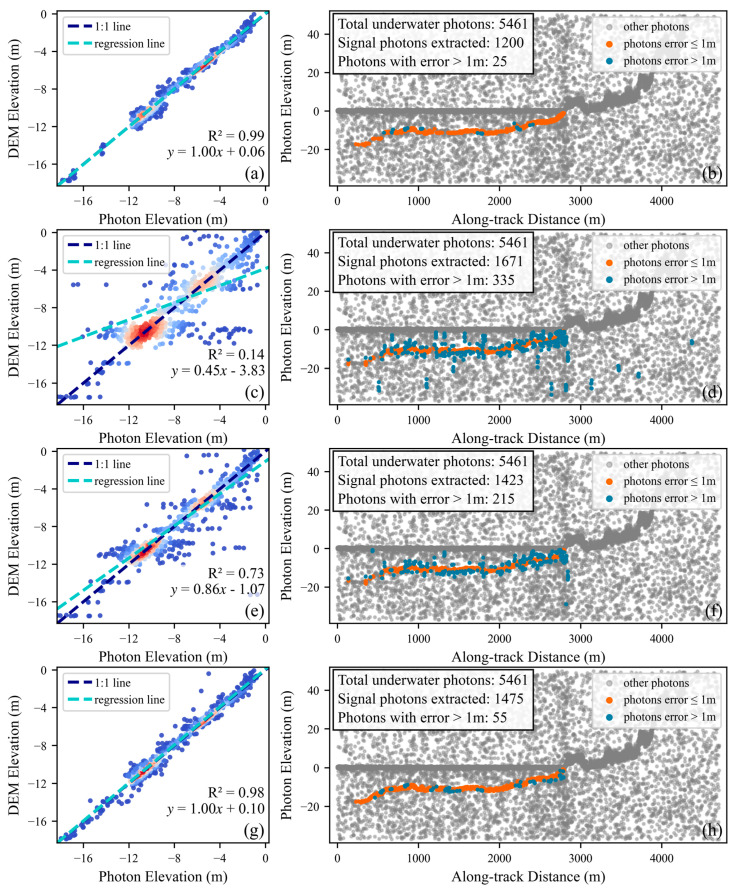
Validation of the three algorithms in area E using underwater signal photons and CUDEM. Panels (**a**,**b**) show ATL24, panels (**c**,**d**) show DBSCAN, panels (**e**,**f**) show the proposed HDWC, and panels (**g**,**h**) show the DNNDA. The blue, white, and red photon colors in panels (**a**,**c**,**e,g**) indicate low, medium, and high density, respectively.

**Figure 12:**
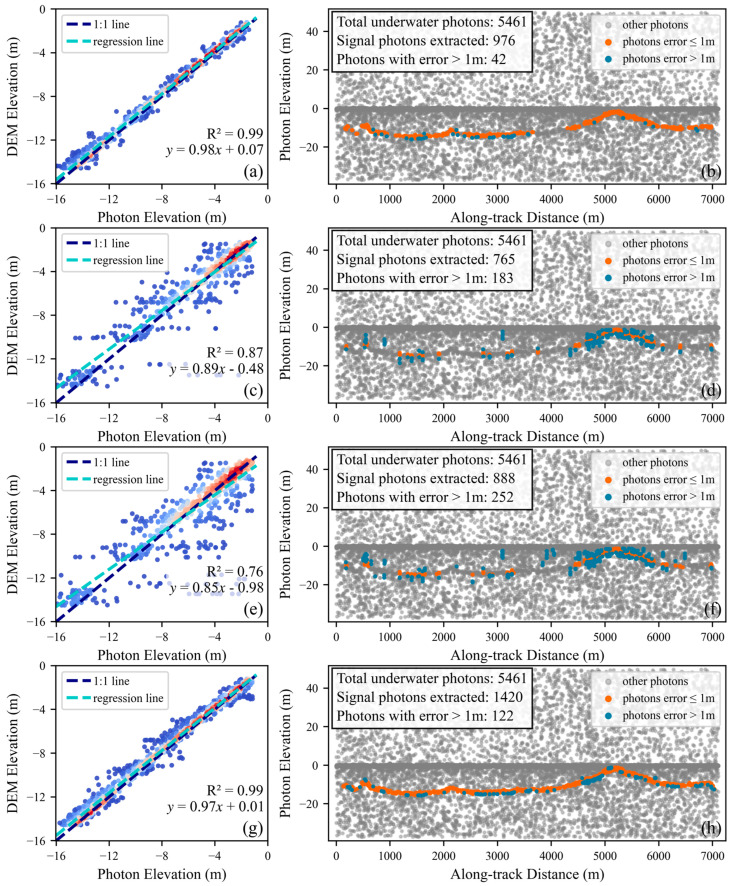
Validation of the three algorithms in area F using underwater signal photons and CUDEM. Panels (**a**,**b**) show ATL24, panels (**c**,**d**) show DBSCAN, panels (**e**,**f**) show the proposed HDWC, and panels (**g**,**h**) show the DNNDA. The blue, white, and red photon colors in panels (**a**,**c**,**e**,**g**) indicate low, medium, and high density, respectively.

Furthermore, we conducted an in-depth analysis of the spatial distribution of extracted signal photons whose elevation errors exceed 1 m relative to the CUDEM dataset, where the error is defined as the absolute difference between the photon elevation and the corresponding CUDEM elevation. In area E (Figure 11), photons with error > 1 m from DBSCAN and HDWC are predominantly distributed along the sides of the signal clusters, indicating a blurred boundary between signal and noise photons in this dataset and making clean separation more challenging. In area F (Figure 12), the extracted signal photons are relatively sparse, with error photons mainly occurring at the water–land interface and as isolated points; these patterns suggest a high background noise rate and reduced distinguishability between signal and noise photons in this area. Nevertheless, the proposed method achieves effective signal extraction across all these scenarios, further confirming its reliability and robustness.

Notably, ATL24 outperforms DNNDA in bathymetric metrics across both areas. Comparing Figure 11 and Figure 12 reveals this advantage stems from ATL24 incorporating fewer signal photons with higher confidence levels that more accurately represent the true seafloor. However, this also results in poorer signal continuity for ATL24.

To address the spatial severity of classification errors, we evaluated the spatial deviation of False Positives (FP-RMSE and FP-MAE) and the spatial deviation of False Negative (FN-RMSE and FN-MAE). These metrics quantify the vertical distance between misclassified photons and the true seafloor profiles in areas E and F.

As demonstrated in Table 5, traditional methods such as DBSCAN and OE not only yield a higher quantity of misclassified signal photons but also exhibit substantial spatial deviations. For instance, in area E, the root-mean-square error of False Positives (FP-RMSE) for DBSCAN reaches 11.67 m. This indicates that its misclassified photons are widely scattered throughout the water, which would severely distort bathymetric inversion. In contrast, the proposed DNNDA significantly minimizes both the occurrence and the spatial magnitude of misclassified signal photons. The FP-RMSE for DNNDA is restricted to only 1.55 m in area E and 1.45 m in area F. This demonstrates that even in the rare cases where DNNDA incurs a classification error, these misclassified photons are tightly constrained within the immediate vicinity of the true seafloor signal band. For misclassified noise photons, the FN-RMSE of all four methods hovers around 0.5 m, consistent with reality since genuine signal photons cluster near the seabed. However, our proposed DNNDA method demonstrates a significantly lower number of misclassified noise photons compared to other approaches, further validating the robustness and engineering reliability of DNNDA for complex, high-noise shallow-water environments.

## 4. Discussion

### 4.1. Effect of Distance Scaling

**Figure 13:**
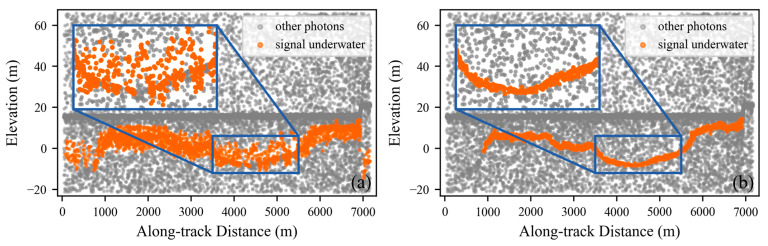
Effect of scale differences on DNNDA. Panel (**a**) shows the original scale. Panel (**b**) shows the scaled result.

This study conducted underwater signal photon extraction experiments using dataset D, with scaling the along-track distance as the control variable. As shown in Figure 13, the zoomed-in views reveal that signal photons extracted after Distance Scaling generally align along the track direction and exhibit slimmer width. Conversely, result obtained without scaling shows a pronounced along-elevation distribution pattern, reduced signal continuity, and broader width. These observations demonstrate that scaling the along-track distance effectively enhances the directional features of neighboring photons, thereby improving the accuracy of signal photon extraction.

### 4.2. Sensitivity Analysis of Parameters

#### 4.2.1. Sensitivity Analysis of Non-Sensitive Parameters

The core steps of this algorithm rely on two non-sensitive parameters: the along-track distance scaling coefficient (default set to 0.025) and the density grade number M (default set to 20). To verify their robustness and ensure they are not overly sensitive to varying environmental conditions, a controlled-variable experiment was conducted across all six datasets (areas A–F). We varied both parameters individually from −60% to +60% of their default values with a step size of 20%. After each adjustment, we identified optimal number of neighboring points k by calculating index in Section 2.2.4 and ultimately assessed extraction performance using F1-scores.

**Figure 14:**
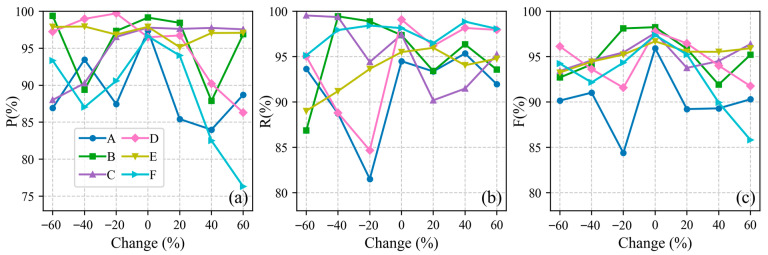
Sensitivity analysis of the along-track distance scaling coefficient. Panel (**a**) shows the precision (P). Panel (**b**) shows the recall (R). Panel (**c**) shows the F1-score (F).

**Figure 15:**
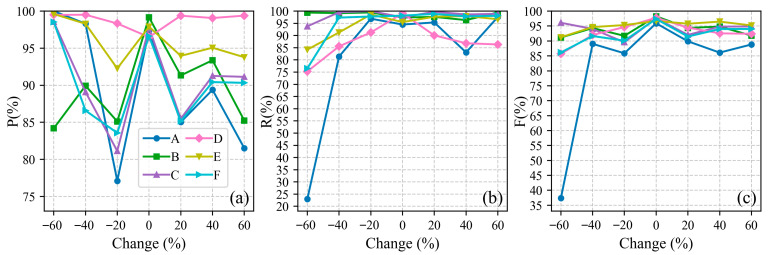
Sensitivity analysis of the density grade number M. Panel (**a**) shows the precision (P). Panel (**b**) shows the recall (R). Panel (**c**) shows the F1-score (F).

Figure 14 and Figure 15 demonstrate that the algorithm exhibits high stability regarding both parameters. For the scaling coefficient, the F1-scores across all six areas remained above 84%, with the vast majority exceeding 90%, regardless of the variations. Similarly, for the density grade number M, the F1-scores generally remained above 85%.

Notably, an exception occurred in area A when M was reduced by −60%, causing the F1-score to plummet below 40%. This is theoretically reasonable: when the density grade number is excessively small, the algorithm fails to capture the fine density gradients between the sparse background noise and the dense signal photons, leading to severe under-segmentation. Overall, these results indicate that within a wide fluctuation range, deviations in the scaling coefficient and M do not significantly degrade the algorithm’s performance, thereby providing a solid theoretical foundation for their default.

#### 4.2.2. Validation of the Optimal Parameter Selection Index

**Figure 16:**
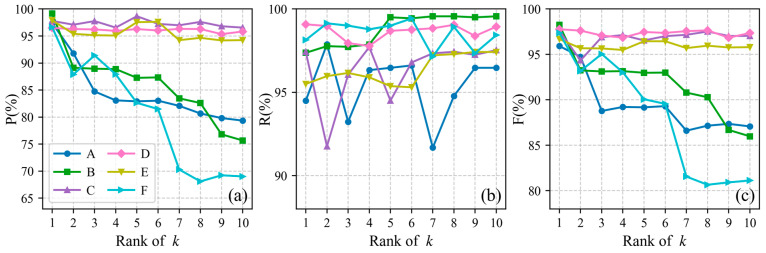
The impact of different ranked values of k on extraction performance. Panel (**a**) shows the precision (P). Panel (**b**) shows the recall (R). Panel (**c**) shows the F1-score (F).

The proposed method contains one sensitive parameter: the number of neighboring points k. To investigate the influence of its variation on extraction performance, and to further verify the effectiveness of the proposed validation index, a controlled-variable experimental design was adopted, in which different values of k were tested. For each dataset, all candidate k corresponding to underwater photons were first sorted in ascending order according to the proposed index, and the top 10 values of k were then selected for signal photon extraction (Table 6). The precision (P), recall (R), and F1-score (F) were computed for each of these 10 settings. Figure 16 illustrates how the extraction performance changes with the ranked parameters, where the parameter corresponding to rank 1 is exactly the one used in Table 2.

Overall, for the six datasets, the precision of signal photon extraction decreases as the rank of the parameter increases, whereas the recall increases. This indicates that parameters with lower ranks tend to extract a larger number of photons at the expense of precision, while higher-ranked parameters yield fewer extracted photons but with higher precision. Notably, for six of the datasets, the highest F1-score is achieved by the parameter at rank 1, which demonstrates that the validation metric proposed in this study can automatically and correctly identify the optimal parameter for each dataset.

### 4.3. Limitations of the Parameter Selection Index and Optimization Strategy

While the parameter-free index demonstrates excellent adaptability and automation in conventional shallow waters, its performance may be challenged in certain extreme scenarios. Theoretically, harsh environmental factors—such as highly turbid water or rough seas—could disrupt the intrinsic photon distribution characteristics, thereby affecting the index’s reliability. Through rigorous data analysis, we identified that complex seabed terrain with inherent signal discontinuities is a definitive boundary condition that can lead to suboptimal parameter selection by the original index.

To explore this limitation and propose a targeted optimization scheme, a highly complex scenario was selected for a detailed case analysis. Specifically, area G utilizes the ATL03 data file ATL03_20231014164258_03922107 (beam gt3l) within the latitude range of [18.11°N, 18.15°N]. Located on the east side of Vieques Island in the Puerto Rico region, this area exhibits dramatic topographic variations and inherent segmented characteristics in the signal photons.

**Figure 17:**
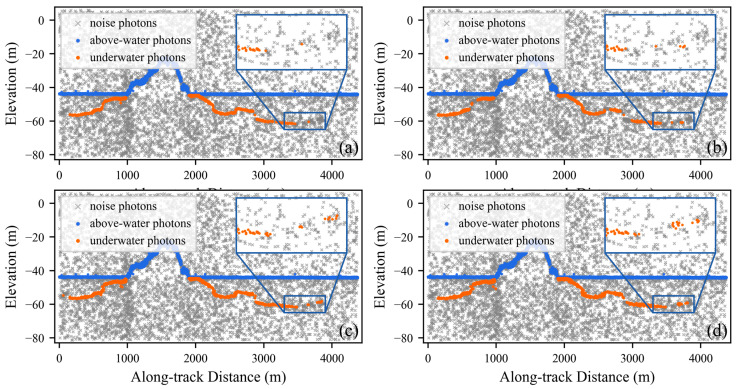
Extraction performance of four distinct k values for underwater signal extraction in area G. Panel (**a**) shows the k = 32. Panel (**b**) shows the k = 88. Panel (**c**) shows the k = 31. Panel (**d**) shows the k = 68.

As shown in Table 7, the original product index identified k=32 as the optimal parameter. While k=32 successfully extracted the primary seabed profile—demonstrating the baseline robustness of the product index—magnified visual inspections (Figure 17a) reveal suboptimal performance in preserving micro-topographic details. Specifically, k=32 fails to extract several inherently discontinuous, yet valid, signal photons at the left terminus. In contrast, experimental validation confirms that k=31 successfully captured these discrete signals, yielding a much more complete terrain representation (Figure 17c).

To understand the underlying cause of this suboptimal selection, we analyze the individual index components. The original multiplication-based model is highly vulnerable to extreme numerical imbalances. For k=32, its ${index}_{2}$ drops to an abnormally low value (0.084), which artificially minimizes the overall product. Consequently, the algorithm over-prioritized extreme local sharpness at the expense of overall signal continuity (${index}_{1}=3486.97$). Thus, truly balanced parameters like k=31, which possess significantly better continuity (${index}_{1}=3171.75$) to capture those discrete signals, are mathematically overlooked.

Therefore, we propose a two-stage robust optimization strategy employing a weighted fusion method to overcome this limitation:

Stage 1: Coarse Screening via Product Index. Utilizing the parameter-free nature of the original product index, we first rank all candidates and extract the Top 20 parameters to form a high-quality candidate pool. This initial step efficiently filters out a massive number of extreme noise configurations.

Stage 2: Fine Selection via Weighted Fusion. Within the refined candidate pool, we apply Min-Max normalization to the components (denoted as ${index}_{1}^{norm}$ and ${index}_{2}^{norm}$) and calculate a newly proposed validation metric:

(21) $${index}^{norm}=w_{1}\cdot index_{1}^{norm}{+w}_{2}\cdot index_{2}^{norm}$$

In complex terrains characterized by inherent discontinuities, maintaining signal completeness is more critical than achieving absolute local sharpness. Therefore, a higher weight is assigned to the continuity index (e.g., $w_{1}=0.8$, $w_{2}=0.2$).

As demonstrated in Table 7, the additive weighted fusion successfully penalizes the extreme imbalance with k=32. Consequently, k=31 and k=68 are automatically re-ranked to the optimal positions. Notably, the ${index}^{norm}$ values for k=31 and k=68 are exceptionally close, indicating highly similar actual denoising performance (Figure 17c,d). This indicates that the weighted fusion metric can identify a region of optimal parameters clusters rather than an isolated, rigid value, thereby enhancing the robustness against parameter sensitivity in complex environments.

In practical applications, we recommend employing the original product index as the default baseline configuration for large-scale, conventional shallow waters due to its high efficiency and parameter-free nature. However, in challenging coastal zones characterized by dramatic topographic relief, extreme turbidity, or inherent signal fragmentation, adaptively switching to this two-stage weighted fusion strategy provides a robust solution to ensure optimal and complete signal extraction.

### 4.4. Detection Capability of the Proposed Method

The proposed DNNDA exhibits strong robustness and high accuracy in underwater signal photon extraction, achieving consistently high precision, recall, and F1-scores across varied geographic settings. In contrast, DBSCAN and HDWC may fail to correctly extract signal photons in data with high background noise, primarily due to their lack of a perception mechanism for directional features in photon distribution. Notably, the ATL24 product also delivers excellent signal photon extraction results across six experimental areas. While it extracts signal photons with higher confidence, this comes at the cost of reduced signal photon continuity compared to DNNDA. In addition, DNNDA shows strong agreement with CUDEM elevations and effective error control, with particularly notable performance in challenging, noise-intensive areas. These results collectively affirm the proposed method’s superior capability in signal photon extraction.

## 5. Conclusions

This study introduces the Directional Nearest Neighbor Distance-based Algorithm, which first compresses the along-track distance of photons to ensure neighboring photons contain more overall scale density features. Subsequently, Under the premise that the density of signal photons in ATL03 data generally exceeds that of noise photons, the algorithm further calculates directional features of the photons. These features are used to adjust the distances to neighboring photons, and the sum of these distances is computed, forming a density representation. Following the refinement of this density representation, an appropriate threshold is determined using the Otsu method to facilitate the extraction of signal photons. Simultaneously, a validation index designed by summarizing signal photon misclassification cases enables more automated determination of sensitivity parameter, further enhancing the adaptability of DNNDA. When compared to manually selected signal photons, the algorithm achieves precision > 96%, recall > 94%, and F1-score > 95% in the extraction of underwater signal photons. Additionally, through regression analysis with high-precision CUDEM data in the Puerto Rico region, the algorithm yields R^2^ > 0.97, RMSE < 0.57 m, and MAE < 0.41 m. Meanwhile, comparisons with ATL24, DBSCAN and HDWC demonstrate that explicitly incorporating directional characteristics of photon distributions leads to a substantial performance gain over conventional isotropic density-based clustering.

These results indicate that integrating scale normalization and directional neighborhood structure modeling yields a more discriminative and stable signal–noise separation than traditional isotropic density measures. DNNDA thus provides an effective framework for satellite-derived bathymetry from photon-counting LiDAR in complex, high-noise shallow-water environments.

## Figures and Tables

**Table 1:** Study areas and corresponding ICESat-2 datasets.

Area	Dataset Name	Beam	Latitude Range (°)
A	ATL03_20181116062310_07430101	gt3r	[16.66, 16.69]
B	ATL03_20200531155706_10100701	gt2l	[22.51, 22.58]
C	ATL03_20201109234112_07150901	gt3l	[12.47, 12.52]
D	ATL03_20181209231549_11050101	gt1r	[21.31, 21.38]
E	ATL03_20230111174702_03391801	gt3r	[18.09, 18.13]
F	ATL03_20210321132920_13371007	gt2r	[18.23, 18.29]

**Table 2:** Adopted values of number of neighboring points for signal photon extraction.

Area	Above Water	Under Water
A	24	22
B	23	20
C	88	37
D	32	73
E	18	72
F	16	33

**Table 3:** Accuracy metrics of underwater signal photons.

Area	ATL24			DBSCAN			HDWC			Proposed DNNDA		
	P (%)	R (%)	F (%)	P (%)	R (%)	F (%)	P (%)	R (%)	F (%)	P (%)	R (%)	F (%)
A	91.52	80.68	85.76	70.29	62.06	65.92	76.41	63.05	69.09	97.38	94.50	95.92
B	89.23	95.20	92.12	78.27	77.47	77.87	81.56	79.66	80.60	99.15	97.37	98.25
C	90.77	94.68	92.69	73.89	73.14	73.51	77.73	60.77	68.21	97.79	97.43	97.61
D	98.04	79.56	87.84	88.45	53.51	66.68	84.65	71.44	77.49	96.49	99.08	97.77
E	98.08	77.84	86.80	78.34	86.57	82.25	83.35	78.44	80.82	97.90	95.5	96.69
F	95.49	66.67	78.52	76.21	41.70	53.91	72.18	45.85	56.08	96.62	98.14	97.37

**Table 4:** Bathymetric accuracy metrics for underwater signal photons.

Area	ATL24			DBSCAN			HDWC			Proposed DNNDA		
	R^2^	RMSE (m)	MAE (m)	R^2^	RMSE (m)	MAE (m)	R^2^	RMSE (m)	MAE (m)	R^2^	RMSE (m)	MAE (m)
E	0.99	0.36	0.27	0.14	5.44	1.45	0.73	1.82	0.68	0.98	0.45	0.31
F	0.99	0.46	0.34	0.87	1.40	0.78	0.76	1.93	1.04	0.99	0.56	0.40

**Table 5:** Bathymetric accuracy metrics for underwater misclassified photons.

Area	Methods	FP-Count	FP-RMSE (m)	FP-MAE (m)	FN-Count	FN-RMSE (m)	FN-MAE (m)
E	ATL24	23	0.97	0.85	335	0.57	0.43
	DBSCAN	362	11.67	5.67	203	0.51	0.39
	HDWC	237	4.37	2.72	326	0.50	0.37
	DNNDA	31	1.55	1.29	68	0.55	0.45
F	ATL24	44	1.09	0.96	466	0.66	0.49
	DBSCAN	182	2.75	2.24	815	0.54	0.40
	HDWC	247	3.58	2.87	757	0.54	0.40
	DNNDA	48	1.45	1.35	26	0.45	0.34

**Table 6:** Top 10 values of k for underwater signal extraction.

Area	Rank_1	Rank_2	Rank_3	Rank_4	Rank_5	Rank_6	Rank_7	Rank_8	Rank_9	Rank_10
A	22	21	61	62	45	44	71	75	67	81
B	20	89	92	90	86	88	97	99	75	87
C	37	20	39	34	33	40	35	36	41	44
D	73	72	80	54	68	67	75	74	57	71
E	72	39	40	41	69	71	44	51	52	50
F	33	27	32	28	35	29	99	88	98	89

**Table 7:** Comparison of parameter selection metrics using the original product index and the proposed weighted fusion strategy in area G.

Parameter (k)	index	Original Rank	$index_{1}$	$index_{2}$	$index_{1}^{norm}$	$index_{2}^{norm}$	$index^{norm}$	New Rank
32	294.88	1	3486.97	0.084	0.266	0.090	0.231	3
88	339.97	2	4141.17	0.082	0.692	0.067	0.567	14
31	465.14	10	3171.75	0.147	0.061	0.673	0.184	1
68	556.21	17	3077.30	0.181	0.000	0.993	0.199	2

## Data Availability

The datasets used in this study include ICESat-2 ATL03 data and CUDEM high-precision terrain data. ICESat-2 ATL03 data can be downloaded from https://search.earthdata.nasa.gov/search, while CUDEM high-precision terrain data can be downloaded from https://coast.noaa.gov/dataviewer/#/lidar/search.
